# First Emergence of Resistance to Macrolides and Tetracycline Identified in *Mannheimia haemolytica* and *Pasteurella multocida* Isolates from Beef Feedlots in Australia

**DOI:** 10.3390/microorganisms9061322

**Published:** 2021-06-17

**Authors:** Tamara Alhamami, Piklu Roy Chowdhury, Nancy Gomes, Mandi Carr, Tania Veltman, Manouchehr Khazandi, Joanne Mollinger, Ania T. Deutscher, Conny Turni, Layla Mahdi, Henrietta Venter, Sam Abraham, Steven P. Djordjevic, Darren J. Trott

**Affiliations:** 1 Australian Centre for Antimicrobial Resistance Ecology, School of Animal and Veterinary Sciences, University of Adelaide, Roseworthy Campus, Roseworthy, SA 5371, Australia; tamara.alhamami@adelaide.edu.au (T.A.); nancy.gomes@student.adelaide.edu.au (N.G.); tania.veltman@adelaide.edu.au (T.V.); khazandi@yahoo.com (M.K.); 2 The ithree Institute, University of Technology Sydney, City Campus, Ultimo, NSW 2007, Australia; piklu.bhattacharya@uts.edu.au (P.R.C.); steven.djordjevic@uts.edu.au (S.P.D.); 3 Department of Animal Health and Production, School of Animal and Veterinary Sciences, University of Adelaide, Roseworthy Campus, Roseworthy, SA 5371, Australia; mandi.carr@adelaide.edu.au; 4 Biosecurity Sciences Laboratory, Department of Agriculture and Fisheries, Coopers Plains, QLD 4108, Australia; joanne.mollinger@daf.qld.gov.au; 5 NSW Department of Primary Industries, Elizabeth Macarthur Agricultural Institute, Menangle, NSW 2568, Australia; ania.deutscher@dpi.nsw.gov.au; 6 Centre for Animal Science, Queensland Alliance for Agriculture and Food Innovation, The University of Queensland, St. Lucia, QLD 4072, Australia; c.turni1@uq.edu.au; 7 Clinical & Health Sciences, University of South Australia, Adelaide, SA 5000, Australia; layla.mahdi@unisa.edu.au (L.M.); rietie.venter@unisa.edu.au (H.V.); 8 Antimicrobial Resistance and Infectious Disease Laboratory, Murdoch University, Murdoch, WA 6150, Australia; s.abraham@murdoch.edu.au

**Keywords:** bovine respiratory disease, antimicrobial susceptibility, *Mannheimia haemolytica*, *Pasteurella multocida*

## Abstract

Bovine respiratory disease (BRD) causes high morbidity and mortality in beef cattle worldwide. Antimicrobial resistance (AMR) monitoring of BRD pathogens is critical to promote appropriate antimicrobial stewardship in veterinary medicine for optimal treatment and control. Here, the susceptibility of *Mannheimia haemolytica* and *Pasteurella multicoda* isolates obtained from BRD clinical cases (deep lung swabs at post-mortem) among feedlots in four Australian states (2014–2019) was determined for 19 antimicrobial agents. The *M. haemolytica* isolates were pan-susceptible to all tested agents apart from a single macrolide-resistant isolate (1/88; 1.1%) from New South Wales (NSW). Much higher frequencies of *P. multocida* isolates were resistant to tetracycline (18/140; 12.9%), tilmicosin (19/140; 13.6%), tulathromycin/gamithromycin (17/140; 12.1%), and ampicillin/penicillin (6/140; 4.6%). Five *P. multocida* isolates (3.6%), all obtained from NSW in 2019, exhibited dual resistance to macrolides and tetracycline, and a further two Queensland isolates from 2019 (1.4%) exhibited a multidrug-resistant phenotype to ampicillin/penicillin, tetracycline, and tilmicosin. Random-amplified polymorphic DNA (RAPD) typing identified a high degree of genetic homogeneity among the *M. haemolytica* isolates, whereas *P. multocida* isolates were more heterogeneous. Illumina whole genome sequencing identified the genes *msr*(E) and *mph*(E)encoding macrolide resistance, *tet*(R)-*tet*(H) or *tet*(Y) encoding tetracycline resistance, and *bla_ROB-1_* encoding ampicillin/penicillin resistance in all isolates exhibiting a corresponding resistant phenotype. The exception was the tilmicosin-resistant, tulathromycin/gamithromycin-susceptible phenotype identified in two Queensland isolates, the genetic basis of which could not be determined. These results confirm the first emergence of AMR in *M. haemolytica* and *P. multocida* from BRD cases in Australia, which should be closely monitored.

## 1. Introduction

Bovine respiratory disease (BRD) is an infectious disease that causes a significant economic loss for the beef industry, accounting for 70–80% morbidity and 40–50% mortality. BRD is a major cause of lost production within the Australian feedlot industry with an estimated annual cost of around AUD 40 million [1,2,3]. BRD is a complex, multifactorial condition that requires the interaction of animals, infectious agents, and environmental factors. Multiple viral and bacterial agents are involved in BRD. Other risk factors include stress from weaning, transport, and marketing, which predisposes calves to infectious agents that cause BRD [4].

The bacteria most often associated with BRD are *Mannheimia haemolytica* and *Pasteurella multocida* [5,6]. *M. haemolytica* and *P. multocida* are both nasopharyngeal commensals of cattle, and after stress or viral infection, they proliferate in the upper respiratory tract and can then migrate to colonise the lungs. BRD is a pneumonic illness ranging from acutely fatal to chronic infections causing permanent lung damage. Acute fibrinous pleuropneumonia is the most common type of pneumonia caused by *M. haemolytica,* while suppurative bronchopneumonia is more typically associated with *P. multocida* [7]. In USA and Canada, *M. haemolytica* was once recognised as the most commonly isolated and economically significant BRD pathogen, responsible for widespread morbidity and mortality [8]. More recently, *P. multocida* has become a more significant BRD pathogen. In one recent Canadian feedlot study, the prevalence of *P. multocida* as a cause of BRD cases was 54.8%, compared to 30.5% for *M. haemolytica* [9].

Management practices employed to reduce BRD incidence include vaccination and antibiotics. Whilst vaccination has demonstrated a moderate protective effect against some agents of BRD, antibiotics still remain the primary management tool for the prevention and treatment of BRD cases. A wide range of antibiotics are used to treat BRD, including β-lactams (most commonly 3rd generation cephalosporins), fluoroquinolones, macrolides, tetracyclines, florfenicol, and sulphonamides [10]. In Australia tetracycline and tilmicosin are recommended to be used as the first line antimicrobial agent for BRD treatment when animals have been found to have clinical symptoms of bacterial infection, where resistance is absent, and tulathromycin is recommended as a second line antimicrobial agent for BRD treatment when a first line drug is ineffective [11]. However, in commercial practice in-field veterinarians often use tulathromycin as a first line therapy due to its superior efficacy with tetracycline used as a second line therapy. The extend-spectrum cephalosporin ceftiofur, which is registered for use in Australia for the treatment of individual cases of BRD, is recommended as a third line treatment for non-responders to first and second lines of treatment or on the basis of susceptibility testing [11]. Fluoroquinolones cannot be used in food-producing animals in Australia [12]. Prompt and effective treatment of BRD reduces clinical signs and decreases fatality rates but is a considerable cost if infection rates are high or if there is a poor response to treatment, for example, because of the development of antimicrobial resistance (AMR) in the target pathogens [13,14].

The agricultural sector is a major end user of antibiotics globally and is one of the key industries where monitoring the development and spread of AMR in bacterial pathogens is critical to maintain their effectiveness. Resistance to antibiotics can be intrinsic, or it may occur by the development of mutations in the bacterial genome, or by the acquisition of AMR genes (ARGs) via horizontal gene transfer. ARGs within multidrug resistance cassettes can be disseminated across bacterial species, and between animals, humans, and the environment [15]. The emergence of AMR associated with BRD pathogens is a significant economic issue affecting the cattle industry globally. Recent studies have identified ARGs clustered within integrative and conjugative elements (ICEs) in *Pasteurellaceae* chromosomes, including those encoding macrolide resistance (*erm*(42), *msr*(E) and *mph*(E)) where they are typically interspersed with ARGs that confer resistance to other drug groups [16,17].

Studies investigating AMR development in BRD pathogens isolated from Australian feedlot cattle are limited, which may in part be due to the low incidence of BRD and the generally good response to treatment observed in individual BRD cases [2,14,18]. Nonetheless, surveillance of AMR in BRD bacterial pathogens is essential to develop treatment protocols that maintain antimicrobial effectiveness, facilitate the implementation of antimicrobial stewardship policies, and improve the health and welfare of animals. Therefore, it is necessary to understand the relationships between AMR phenotype and genotype in BRD bacterial pathogens as well as assess the role of antibiotic use in the development and spread of ARGs. The current study evaluated the AMR profiles of *M. haemolytica* and *P. multocida* isolates obtained from the lungs of necropsied cattle at feedlots in four Australian states over a five-year period (2014–2019). The molecular epidemiology of these isolates was characterised by RAPD-PCR. Furthermore, whole-genome sequencing (WGS) was undertaken to identify ARGs and confirm correlation between AMR phenotype and genotype.

## 2. Materials and Methods

### 2.1. Sample Collection and Isolation Procedures:

A total of 228 isolates (88 *M. haemolytica*; 140 *P. multocida*) were obtained between 2014 and 2019 from post-mortem samples from BRD-affected cattle submitted to Veterinary Diagnostic Laboratories servicing the beef feedlot industry in Queensland (QLD), New South Wales (NSW), Victoria (VIC), and South Australia (SA) [19]. Eleven large Australian feedlots (three in Qld, four in NSW, one in VIC, and three in SA) were encouraged to submit samples in 2019 during BRD peak case occurrence (typically at the start of autumn and spring). Samples submitted included aseptically collected deep lung swabs, lung and heart (if grossly affected) tissue samples and pericardial, pleural or peritoneal fluid (if present). All collected samples could be traced to individual animals and related clinical data including details of any antimicrobial therapy initiated. All swabs were placed in Amies^®^ bacteria transport media. Samples were stored at 4 °C until delivered to the referring veterinary diagnostic laboratory where microbial inoculation was performed generally within >48 h of collection. Each Veterinary Diagnostic Laboratory followed their standard operating procedures for isolation (i.e., Sheep Blood Agar, MacConkey Agar and Chocolate Agar) incubated under CO_2_ enriched conditions, and identification of BRD pathogens. Individual colonies of suspicious microbial growth were subcultured to obtain pure growth for microbial identification using MALDI-TOF Mass Spectroscopy (Bruker, Preston, VIC, Australia) and/or biochemical testing.

### 2.2. Isolate Storage and Transfer

Isolates were periodically sent to the ACARE Antimicrobial Resistance Laboratory in SA on Amies^®^ transport media for antimicrobial susceptibility testing. The breakdown of isolates by year is shown in Table 1. Swabs were cultured within 48 h of receipt onto Sheep blood agar (SBA) plates and incubated aerobically at 37 °C for 24 h. Microbial growth on each plate was assessed to confirm purity and the identity was re-confirmed by MALDI-TOF. All *P. multocida* and *M. haemolytica* pure cultures were stored in Trypticase Soy Broth with 20% (*w*/*v*) glycerol at −80 °C for subsequent antimicrobial susceptibility testing.

### 2.3. Antimicrobial Susceptibility Testing

Minimum Inhibitory Concentration (MIC) antimicrobial susceptibility testing was performed by broth microdilution using Veterinary Reference Card panels (Sensititre^®^, Trek Diagnostics, ThermoFisher Scientific, Thebarton, South Australia), specifically, the Bovine BOP07F panel. The Thermo Scientific™ Sensititre™ SWIN™ Software System was used to interpret the MIC values manually using a Sensititre VizionTM viewing system. The data system uses CLSI breakpoint recommendations, but they are not veterinary based. Therefore, MIC values were manually interpreted for antimicrobials used in veterinary medicine by adopting CLSI recommended veterinary breakpoints [20].

Nineteen antimicrobials were tested: ampicillin, ceftiofur, clindamycin, danofloxacin, enrofloxacin, florfenicol, gamithromycin, gentamicin, neomycin, tetracycline, penicillin, sulphadimethoxine, spectinomycin, tiamulin, tilmicosin, trimethoprim/sulfamethoxazole, tulathromycin, tylosin tartrate, and tildipirosin. The majority of these antimicrobials are currently registered for use in food-producing animals in the US and Canada to treat BRD as well as other cattle infections in cattle [21]. However, in Australia ceftiofur, chlortetracycline, oxytetracycline, tilmicosin and tulathromycin are the most commonly used antimicrobials to treat BRD infection in feedlot cattle [22,23]. Control reference strains included *S. aureus ATCC* 29213, *M. haemolytica ATCC* 33396, *E. coli ATCC* 25922, and *E. coli ATCC* 35218. Breakpoints are listed in Table 2.

*P. multocida* and *M. haemolytica* isolates were subcultured onto Tryptic Soy Agar containing 5% (*w*/*v*) Sheep blood (TSA-B) and incubated at 36 ± 1 °C. Direct colony suspension was used to prepare the bacterial inoculum equivalent to a 0.5 McFarland Standard, using 5 mL normal saline. A 10 µL aliquot of the suspension was transferred into a tube of 11 mL Sensititre Mueller-Hinton broth to give an inoculum of 1 × 10^5^ cfu/mL. After vortexing, the Sensititre plate was inoculated, plates were sealed with seal strips and incubated at 35 °C for 18 h. The MICs were interpreted, and MIC_50_, MIC_90_, MIC range, % resistant and % multidrug-resistant (resistant to one or more antimicrobials in three or more classes) for each bacterial species determined [24]. The frequency of resistance for each antimicrobial agent was described as rare: <0.1%; very low: 0.1% to 1.0%; low: >1.0% to 10.0%; moderate: >10.0% to 20.0%; high: >20.0% to 50.0%; very high: >50.0% to 70.0%; and extremely high: >70.0%; according to the European Food Safety Authority (EFSA) and the European Centre for Disease Prevention and Control [25].

### 2.4. Molecular Typing-RAPD

Random Amplified Polymorphic DNA (RAPD) amplification was performed using ERIC-1026 (5′-TACATTCGAGGACCCCTAAGTG-3′) primer to determine genetic differences among *P. multocida* isolates [26] and OPG-13 (5′-CTCTCCGCCA-3′) (Sigma-ALDRICH) for *M. haemolytica* with some modifications [3]. Briefly, DNA was extracted using the boiled lysate method [27], and the RAPD PCR was performed in a final reaction volume of 25 μL with MyTaq HS Red Mix (Bioline), 100 ng of template DNA, and 0.4 μM of primer. The reaction mixtures were run on a thermal cycler (Bio-Rad) with cycling conditions for ERIC-1026 commencing with an initial 2 cycles of 5 min each at 95 °C, 35 °C, and 72 °C, followed by 31 cycles of 1 min at 95 °C, 1 min at 60 °C, and 2 min at 72 °C, with a final extension at 72 °C for 8 min. Meanwhile, OPG-13 amplification cycles commenced at 95 °C for 1 min, 35 °C for 30 s, and 72 °C for 1 min for the first cycle; followed by 95 °C for 40 s, 35 °C for 30 s, and 72 °C for 1 min for 39 cycles, with a final extension at 72 °C for 10 min. The RAPD PCR products were separated by electrophoresis on a 2% (*w*/*v*) agarose gel in 1× TAE (Tris-acetate-EDTA) buffer and SYBR DNA Gel Stain (Invitrogen) at 100 V for 2 h, with a 1 Kb molecular weight ladder (Bioline). The RAPD-PCR banding patterns were analysed using BioNumerics, Version 7.6 (Applied Maths).

### 2.5. Whole Genome Sequencing and Analysis

All 88 *M. haemolytica* isolates and 119 of the 140 *P. multocida* isolates, representing the major sub-clusters identified in the RAPD dendrogram and including all isolates exhibiting resistance to one or more antimicrobials, were selected for whole-genome sequencing for resistance genotype interrogation. DNA was extracted from late-log phase cultures growing in Brain-heart infusion broth for 6 h, using the Isolate II Genomic DNA kit from Bioline and following manufacturer’s instructions. Paired end (150 nt) sequencing libraries were prepared using the published Hackflex protocol [28]. Genomes were sequenced on the Illumina HiSeq sequencing HiSeq sequencing platform, and raw reads were assembled using Shovill (https://github.com/tseemann/shovill (accessed 5 December 2019), the de novo assembly protocol. To evaluate presence of genes corresponding to the AMR profile of isolates in the genomes, a targeted gene search approach was adopted using BLASTn function and an internal database of genes reported to have contributed towards the respective resistance phenotypes in *Pasteurella* and *Mannheimia* genomes on the High-Power Computing cluster available at the University of Technology Sydney. BLASTn outputs were filtered for hits that returned ≥98% sequence identity across 100% of the input query length. For genomes where genotypes could not be matched with the phenotypes, ResFinder database was used to identity resistance genes.

### 2.6. Data Analysis

Based on accessible CLSI veterinary breakpoints (Table 2), MICs for each antimicrobial were summarised and recorded as susceptible, intermediate or resistant [20]. CLSI veterinary breakpoints for clindamycin, gentamycin, neomycin, sulphadimethoxine, tiamulin, trimethoprim/sulfamethoxazole, and tylosin tartrate, have not been established for either microbial species investigated and therefore these could not be interpreted. Resistance percentages were calculated by dividing the number of resistant isolates over the total number of isolates for two sampling periods (2014–2018 and 2019). Confidence intervals were calculated using the method described by Wilson [29].

## 3. Results

### 3.1. MIC Distribution of M. Haemolytica and P. Multocida

The majority of samples were obtained from animals that had been treated with antibiotics and were euthanised due to poor response to treatment (i.e., untreated pen deaths were uncommon events). Obtaining multiple pathogens from single sample submissions was a common feature and a high BRD pathogen isolation rate was obtained (>90%) with *Histophilus somni*, *Mycoplasma* spp., *Bibersteinia trehalosi* and *Trueperella pyogenes* isolates stored for subsequent analysis (data not shown). All *P. multocida* isolates (*n* = 140) were susceptible to ceftiofur, danofloxacin, enrofloxacin, florfenicol, spectinomycin, and tildipirosin. The *M. haemolytica* isolates (*n* = 88) were pan-susceptible to all antimicrobials tested that had CLSI breakpoints available except for a single isolate from the 2019 collection that was resistant to gamithromycin, tilmicosin, and tulathromycin (Table 3 and Table 4). This *M. haemolytica* (19BRD-084) isolate was obtained from the same lung sample as a *P. multocida* isolate exhibiting the same phenotype.

The 75 *P. multocida* isolates from 2014–2018 showed a low level of resistance to ampicillin/penicillin (4%), gamithromycin (2.7%), tetracycline (8.0%), tilmicosin (2.7%), and tulathromycin (2.7%) (Table 5). Among the 65 *P. multocida* isolates obtained in 2019, resistance prevalence was high for gamithromycin (21.5%), tilmicosin (24.6%), and tulathromycin (21.5%), moderate for tetracycline (18.5%), and low for ampicillin/penicillin (4.6%) (Table 5 and Table 6).

### 3.2. Isolates from 2014–2015

**Figure 1:**
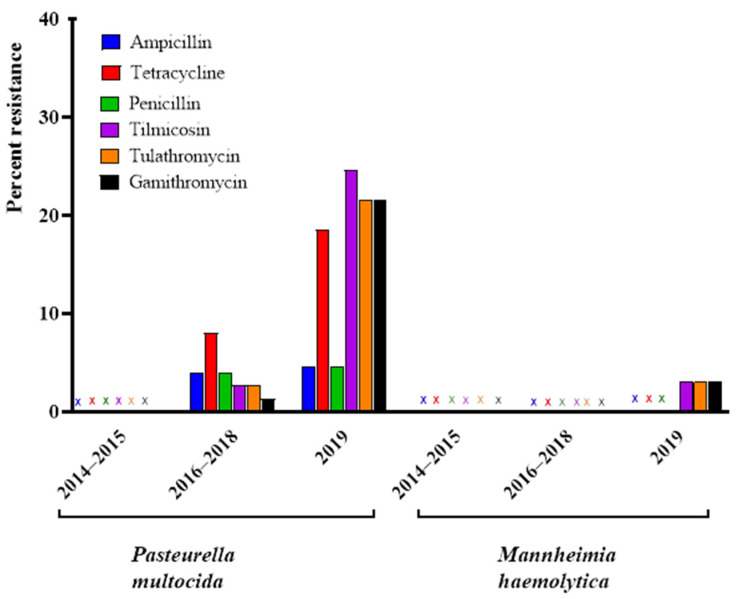
Percentage of *Pasteurella multocida* (*n* = 140) and *Mannheimia haemolytica* (*n* = 88) isolates classified as resistant to selected antimicrobials determined by antimicrobial susceptibility testing over the 2014–2019 collection period, (X) signifies no resistance observed.

All P. multocida (*n* = 12) and M. haemolytica (*n* = 11) isolates from 2014–2015 were susceptible to all antimicrobials tested that had CLSI breakpoints available (Figure 1).

### 3.3. Comparison of Resistance Profiles from 2016–2019 Reveals an Increase in Resistant Isolates over Time

Among the *P. multocida* isolates, the prevalence of resistance to macrolides and tetracycline increased over time. The first detection of resistance was observed in 2016–2017 isolates, with four exhibiting resistance to tetracycline, one of which was also resistant to aminopenicillins, and a single isolate found to be resistant to tilmicosin, tulathromycin, and gamithromycin.

In 2018, only two isolates were resistant to tetracycline, one of which had the aminopenicillin resistance phenotype. Another isolate was resistant to aminopenicillins only, and a single isolate was resistant to all three macrolides (gamithromycin, tilmicosin, and tulathromycin). In 2019, a markedly higher proportion of isolates exhibited resistance to macrolides and tetracycline. A total of ten isolates were resistant to macrolides only (gamithromycin, tilmicosin, and tulathromycin). A further four isolates were resistant to tetracycline only while five isolates exhibited dual resistance to all the macrolides as well as and tetracycline. An additional two isolates exhibited the aminopenicillin-tetracycline-tilmicosin resistance phenotype, the first identified multi-drug resistant (MDR) isolates in the collection. A single isolate exhibited the aminopenicillin-tetracycline resistance phenotype previously identified in the 2016–2018 isolates (Table 7 and Figure 1).

### 3.4. Phylogenetic Analysis Using RAPD

RAPD analysis was performed in order to investigate the phylogeny of the isolates from different Australian states. The 88 *M. haemolytica* isolates were separated into 12 main clusters. Clusters *VI* and *X* contained isolates from three states (NSW, QLD, and SA) (26/88 and 12/88, respectively). Three clusters contained isolates from both NSW and QLD (Clusters *I/III*, and *VII),* with the sole resistant isolate located in Cluster *III* (1/4; 25%). Clusters *II/IV*, and *V* consisted predominately of QLD isolates. Clusters *XI* and *XII* contained VIC and NSW isolates, respectively (Figure S1).

RAPD analysis of the 140 *P. multocida* isolates identified 10 clusters. Cluster *I/II,* and *V* contained isolates from the four studied states. Three clusters contained isolates from both QLD and NSW (clusters *IV/VIII*, and *IX*), followed by cluster *III* and *VI*, which consisted predominately of QLD isolates (5/15 and 17/28, respectively). Clusters *VII* and *X* contained isolates exclusively from QLD (two and five isolates, respectively) (Figure S2, Table 8). WGS was undertaken on all isolates per cluster, including isolates exhibiting resistance to one or more antimicrobials or obtained from distinct feedlots in different Australian states of origin.

### 3.5. Correlating Phenotypic Resistance with Genotypic Resistance Elements Using Whole Genome Sequencing (WGS)

The genomes of all 88 *M. haemolytica* and 119 of the 140 *P. multocida* isolates were sequenced to identify ARGs, and to collect preliminary information on their context. The *tet*(H)*-tet*(R) ARGs encoding a multidrug efflux pump were present in several of the isolates that exhibited tetracycline resistance, and five of these isolates possessed the *bla_ROB-1_* β-lactamase ARG. An additional isolate had a tetracycline MIC of 4 µg/mL (intermediate value) but still carried the *tet*(H)*-tet*(R) ARGs (19BRD-111). A less common tetracycline ARG identified was the *tet*(Y) tetracycline ARG, which was always found associated with kanamycin/neomycin (*aphA1*), streptomycin (*str*A, *str*B-like), and sulfonamide (*sul*2) ARGs. Thirteen isolates including the single macrolide-resistant *M. haemolytica* isolate contained the macrolide ARGs *msr*(E) and *mph*(E) and three of five isolates exhibiting dual resistance to macrolides and tetracyclines were confirmed to contain both *tet*(H)-*tet*(R) and *msr*(E)*/mph*(E) ARGs. Two *P. multocida* isolates (19BRD-110 and 19BRD-146) were resistant to macrolides but did not contain any of the known macrolide resistance genes (Table 8). Most *P. multocida* isolates from 2016–2018 that contained ARGs (i.e *aphA1, bla_ROB-1_*, *strA, strB, tet*(Y), *tet*(H)*-tet*(R), *sul2, msr*(E), and *mph*(E)) belonged to cluster V (9/12, 75%). By contrast, 21 *P. multocida* isolates from 2019 that contained ARGs *tet*(H)*-tet*(R), *bla_ROB-1_, msr*(E)*,* and *mph*(E) were distributed into clusters *IV* (2/22; 9.1%), *VI* (8/28; 28.6%), *VII* (1/2; 50%), *VIII* (7/13; 53.8%), and *IX* (3/7; 42.9%) (Table 8). 

## 4. Discussion

This study reports the first identification of resistance among Australian isolates of *M. haemolytica* and *P. multocida* associated with fatal cases of BRD in Australian feedlot cattle. The major findings from this study were: (1) The proportion of cases involving *P. multocida* in the aetiology was considerably higher in comparison to *M. haemolytica*-associated cases and *P. multocida* isolates were more genetically diverse; (2) A significant proportion of *P. multocida* isolates (31/140; 22.1%) expressed resistance to at least one antimicrobial class (macrolides, tetracycline, or ampicillin/penicillin) or combinations thereof, with a higher prevalence of resistance in more recent (i.e., 2019) isolates; (3) WGS identified the genes responsible for resistance to aminopenicillins (*bla_ROB-1_*), macrolides *(msr*(E) and *mph*(E)), and tetracycline (*tet*(H)*-tet*(R)*)* in *P. multocida;* (4) There was only a single *M. haemolytica* isolate resistant to macrolides identified, which also possessed *msr*(E)-*mph*(E) genes and was isolated from the same animal that yielded a macrolide-resistant *P. multocida* isolate.

In this study, obtaining aseptically collected samples from pneumonic lung tissue of autopsied animals yielded a high proportion of BRD pathogens (data not shown) often as mixed infections, given that submissions for culture and susceptibility testing included two swabs obtained from different lung lobes together with a fresh pneumonic lung tissue sample. Moreover, *P. multocida* was the most prevalent pathogen identified in Australian feedlot cattle affected by BRD (data not shown), particularly in comparison to *M. haemolytica.* This could possibly be related to *P. multocida*’s ability to inhibit the growth of *M. haemolytica* [30], but more likely reflects the fact that a high proportion of feedlot cattle in the study were vaccinated with Bovilis MH^TM^ (Intervet) [31], a highly efficacious *M. haemolytica* vaccine developed in Australia [23,32]. Contrary to these findings, *M. haemolytica* had a 91% prevalence in a recent North American BRD study, with only 8% prevalence reported for *P. multocida* [33]. However, another North American study also found that *P. multocida* was the most commonly identified pathogen in BRD-affected cattle (54.8%), followed by *M. haemolytica* (30.5%), but the isolates in this study were obtained by sampling live animals (via transtracheal wash) as opposed to post-mortems conducted on dead cattle [9]. Some additional differences between North American and Australian feedlots that may explain the disparity in BRD pathogen prevalence include variable vaccine efficacy, age at induction, length of time on feed, lower stocking densities, and different environmental conditions [14].

A significant proportion of *P. multocida* isolates (12/140; 8.6%), the majority of which were isolated in 2019, exhibited resistance to the macrolides gamithromycin, tilmicosin, and tulathromycin. Smaller proportions exhibiting resistance to tetracycline only (8/140; 5.7%), dual resistance to both macrolides and tetracycline (5/140; 3.6%), or aminopenicillins and tetracycline (3/140; 2.1%), and multidrug resistance to aminopenicillins, tetracycline, and tilmicosin (2/140; 1.4%). Resistant isolates were widely distributed throughout Australian feedlots and represent a significant future risk to the industry, considering that macrolides (tilmicosin and tulathromycin only, as gamithromycin and tildipirosin are not yet registered in Australia) and oxytetracycline are the most commonly prescribed injectable antimicrobial agents for cases of BRD, with ceftiofur considered a reserve agent [34]. The preference for macrolides may in part be due to their significant anti-inflammatory and immunomodulatory effects [34]. A recent systematic review has also identified that macrolides are the most effective antibiotics for reducing the incidence of BRD when used within the first 45 days of feedlot entry [35]. A previous study noted that resistance to antimicrobials used in the treatment of BRD in Australian feedlots was uncommon, however, it is important to highlight the limited number of AMR studies undertaken in Australia prior to the present study [2,18,36].

The increased rate of resistance in *P. multocida* isolates obtained in 2019 compared to previous years suggests that resistance has only recently emerged in Australia, or that increased sampling has identified a previously undetected reservoir of resistance. When antimicrobials such as macrolides, oxytetracycline and chlortetracycline are used for both treatment and metaphylaxis, resistant organisms are more likely to be detected [9,37]. We observed significantly lower levels of resistance in BRD pathogens from Australian feedlot cattle compared to recent North American studies. In one Canadian study, the majority of BRD isolates (90.2%) were resistant to at least one of the three macrolides tested (tilmicosin, tulathromycin, and tylosin) [38].

In the present study, *M. haemolytica* was confirmed to be fully susceptible to all licensed antibiotics for BRD treatment, except for a single isolate resistant to the three macrolides gamithromycin, tilmicosin and tulathromycin (1/88; 1.1%). This low rate of resistance is considerably below that of many international studies. Large-scale surveillance programmes in BRD pathogens in the United States and Canada over a 10-year period (2000–2009) showed a progressive increase in *M. haemolytica* resistance to tilmicosin and tulathromycin [21]. More recent studies have documented resistance to macrolides in > 75% of *M. haemolytica* isolates from United States cattle between 2013–2015 [39]. By contrast, surveys undertaken in European cattle (2009–2012), have observed generally low rates of resistance to macrolides (0–4.0%) and tetracycline (3.0–12.0%) in *M. haemolytica* [40].

RAPD-PCR has been an effective tool for epidemiological typing of both *P. multocida* and *M. haemolytica* [41,42,43]. In the present study, the RAPD-PCR verified that the Australian *M. haemolytica* isolate collection was genetically homogeneous. However, the confidence score around the deeper nodes in the cladogram are fairly low, indicating that clusters *I–XII* are likely sub-clusters of a single major cluster. We revisited the banding patterns in the gel and identified a number of prominent bands that appear consistently across all isolates in clusters *I–XII.* By contrast, the *P. multocida* collection was extremely diverse. This was in line with a previous study that demonstrated a substantial degree of genetic variation between *P. multocida* isolates from a wide range of host species, even among isolates from the same animal or geographic origin [44].

Another study suggested that Australian *P. multocida* isolates from sporadic outbreaks of porcine pneumonia are non-toxigenic (*toxA-*) and display heterogeneous DNA restriction *endonuclease* profiles compared with toxigenic isolates from herds with progressive atrophic rhinitis [45]. Contrary to these findings, another study identified considerably less diversity among *P. multocida* isolates obtained from cases of BRD [46]. This dichotomy raises questions about the reliability of RAPD in assessing genetic relatedness, in particular for *Mannheimia,* and highlights the necessity of WGS approaches for better resolution of phylogenetic relationships among BRD pathogens in Australia, which is currently underway.

Preliminary WGS analysis identified nine ARGs in resistant *P. multocida* and *M. haemolytica* isolates encoding resistance to streptomycin (*str*A and *str*B), tetracycline *(tet*(H)-*tet*(R) and *tet*(Y)), macrolides (*msr*(E), *mph*(E)), neomycin/kanamycin (*aphA1)*, sulfonamide (*sul2*) and *β*-lactams (*bla_ROB-1_*). Moreover, the resistant *P. multocida* strains shown to contain known ARGs appear to be genetically related by RAPD-PCR. *P. multocida* and the single macrolide-resistant *M. haemolytica* isolate containing *msr*(E) and *mph*(E) had high MICs for gamithromycin and tulthromycin, and low MICs for tildiprosin, whereas *erm*(42) (not detected in our study) imparts high clindamycin and tildipirosin MICs [47].

Macrolide (*msr*(E)*, mph*(E)) and tetracycline ARGs (*tet*(H)*-tet*(R)) had a relatively high prevalence in the *P. multocida* collection. Moreover, it is possible that the single macrolide-resistant *M. haemolytica* strain may have acquired the *msr*(E) and *mph*(E) ARGs through horizontal transfer from *P. multocida* [33,48]. Deeper whole-genome sequence analysis will be required to confirm the genetic context of these genes and their potential transmissibility. Other researchers have noted these ARGs have dramatically increased in BRD isolates in recent years where they are mainly associated with integrative conjugative elements (ICE) [49]. Furthermore, *tet*(H)-*tet*(R) has been detected on plasmids and in chromosomal DNA in BRD isolates. Interestingly, this study is the first to describe the tetracycline efflux pump gene tet(Y) in *P. multocida* isolates which may be possibly co-located with streptomycin (*strA*-*strB*), neomycin/kanamycin (*aphA1)* and sulfonamide (*sul2*) ARGs within a mobile genetic element. Despite the fact that resistance caused by the *tet*(Y) gene has not yet been well described in the *Pasteurellaceae* family [50], *tet*(Y) has been found in *Acinetobacter, Escherichia, Pelosinus,* and *Rhizobium* bacterial genera [51,52,53].

Further studies are required to describe the genetic context of ARGs identified in two linked QLD *P. multocida* isolates exhibiting the unusual resistance phenotype (aminopenicillin-tetracycline-tilmicosin resistance) and shown to contain only *tet*(H)-*tet*(R) and the β-lactamase *bla_ROB-1_* ARGs. The *bla_ROB-1_* gene has been described in plasmids carried by isolates of *P. multocida* and *Haemophilus parasuis* recovered from animals in Spain [54,55]. Recently, the *bla_ROB-1_* gene has also been reported in *Haemophilus influenzae* clinical isolates of human origin [56]. Subsequently, Europe and USA studies have revealed that the *bla_ROB-1_* gene is often located within ICE present in BRD isolates [5,57]. The resistance to tilmicosin in these MDR isolates could potentially be attributed to point mutations in the 23S rRNA gene which have been correlated with high level macrolide resistance (MICs > 64 µg/L), in other members of the *Pasteurellaceae* family [49], or another currently unknown mechanism. Further interrogation of the whole genome sequence data will be required to confirm this.

This study had some limitations. Diagnostic samples were all obtained at post-mortem from animals that had died (a minority) or were euthanised due to BRD that was non-responsive to antibiotics (the majority). As such, the presence of resistant isolates may be one of the reasons (but not the only one) for treatment failure. Ideally, samples would be obtained from live animals (via transtracheal wash) prior to antibiotic treatment to obtain a true resistance prevalence rate estimation among feedlots. However, this would require veterinary intervention where our project was designed so that feedlot animal health workers could undertake the sampling at post-mortem, given the large distances between feedlots in Australia and the infrequency of veterinary visits (approximately once per month). Secondly, antimicrobial resistance phenotypes and genotypes were not 100% correlated and potentially new mechanisms of resistance (such as tilmicosin resistance in QLD *P. multocida* isolates) and/or gene mutations could not be adequately screened for in this preliminary analysis. Lastly, it was not possible using Illumina HiSeq sequencing to determine the genetic context of the ARGs and their possible co-location within plasmids or ICE.

## 5. Conclusions

This publication aims to provide a comprehensive analysis of the antimicrobial susceptibility of BRD pathogens *M. haemolytica* and *P. multocida* isolated from feedlot cattle in Australia from 2014 to 2019 and their association with identified ARGs. This knowledge can guide clinicians in their choice of therapy for Australian cattle, whilst reinforcing the principle of aseptic sampling techniques at post-mortem for BRD affected cattle, and the importance of WGS to correlate phenotypic resistance with possible genetic determinants among *P. multocida* and *M. haemolytica* isolates. Our overall interpretation of data suggests an emerging resistance to macrolides and tetracyclines identified in *P. multocida* isolates and a single *M. haemolytica.* The RAPD assay used previously for epidemiological studies to evaluate *P. multocida* and *M. haemolytica* relatedness is an appropriate screening tool prior to further detailed analysis by WGS. The RAPD assay revealed that *P. multocida* isolates from Australian feedlot cattle were genetically heterogeneous. In this study, WGS of isolates identified ARGs that are responsible for the antimicrobial resistance phenotype and provides an early indication of possible mobile genetic elements (plasmids or ICE) present in feedlot BRD isolates which warrants further investigation. Most of the isolates were obtained from animals affected by BRD that had a history of antimicrobial therapy, suggesting possible treatment failure due to resistance in the causal pathogens. Continued monitoring is needed to support antimicrobial stewardship programs recently developed by the industry designed to ensure the effectiveness of veterinary antimicrobial drugs.

## Figures and Tables

**Table 1:** Major bovine respiratory disease isolates obtained in the 2014–2019 collection period.

Isolates	2014/2015	2016/2017	2018	2019	Total
*Mannheimia haemolytica*	11	23	21	33	88
*Pasteurella multocida*	12	40	23	65	140
Total	23	63	44	98	228

**Table 2:** Summary table of CLSI veterinary clinical MIC breakpoints used for BRD isolates.

Antimicrobial Agent	MIC Breakpoint (µg/mL)		
	Susceptible	Intermediate	Resistant
Ampicillin	≤0.03	0.06–0.12	≥0.25
Ceftiofur	≤2	4	≥8
^a^ Clindamycin	-	-	-
Danofloxacin	≤0.25	0.5	≥1
Enrofloxacin	≤0.25	0.5–1	≥2
Florfenicol	≤2	4	≥8
Gentamicin	-	-	-
Gamithromycin	≤4	8	≥16
Neomycin	-	-	-
Penicillin	≤0.25	0.5	≥1
Sulphadimethoxine	-	-	-
^b^ Specinomycin	≤32	64	≥128
Tetracycline	≤2	4	≥8
Tiamulin	-	-	-
^c^ Tilmicosin	≤8	16	≥32
Trimethoprim/sulfamethoxazole	-	-	-
Tulathromycin	≤16	32	≥64
Tylosin tartrate	-	-	-
^d^ Tildipirosin	≤8	16	≥32

MIC breakpoints are taken from Clinical and Laboratory Standards Institute [20]. ^a^ For clindamycin, gentamicin, neomycin, tiamulin, trimethoprim/sulfamethoxazole, and tylosin tartrate, no CLSI breakpoints are available for cattle. ^b^ For specinomycin, CLSI breakpoints for cattle only validated for *P. multocida* were used. ^c^ For tilmicosin, CLSI breakpoints for cattle only validated for *M. haemolytica were used*. ^d^ For tildipirosin, in *M. haemolytica* the CLSI breakpoints were S ≤ 4, I = 8 and R ≥ 16.

**Table 3:** MIC distribution frequencies of *M. haemolytica* cattle isolates from Australia 2014–2018.

	*Mannheimia haemolytica* (*n* = 55)																	
	MIC Distribution (µg/mL) ^a^															MIC_50_ (µg/mL)	MIC_90_ (µg/mL)	CI (95%) ^b^
Antibiotics	0.03	0.06	0.12	0.25	0.5	1	2	4	8	16	32	64	128	256	512			
Ampicillin				100	0	0	0	0	0	0						0.25	0.25	0–8.13
Ceftiofur				100	0	0	0	0	0							0.25	0.25	0–8.13
Clindamycin				0	0	1.8	1.8	0	80.0	14.5	1.8					8	16	0–8.13
Danofloxacin			98.2	0	1.8	0										0.12	0.12	0–8.13
Enrofloxacin			98.2	0	0	1.8	0									0.12	0.12	0–8.13
Florfenicol				7.3	89.1	3.6	0	0	0							0.5	1	0–8.13
Gamithromycin						98.2	1.8	0	0							1	1	0–8.13
Gentamicin						3.6	94.5	1.8	0	0						2	4	0–8.13
Neomycin								30.9	69.1	0	0					4	8	0–8.13
Tetracycline					98.2	1.8	0	0	0							0.5	1	0–8.13
Penicillin			47.3	43.6	9.1	0	0	0	0							0.12	0.5	0–8.13
Sulphadimethoxine														100		256	256	0–8.13
Spectinomycin									0	20.0	78.2	1.8				16	32	0–8.13
Tiamulin					0	1.8	1.8	0	12.7	80.0	3.6					8	16	0–8.13
Tilmicosin							0	45.5	50.9	3.6						4	8	0–8.13
Trimethoprim/ sulfamethoxazole							100									2	2	0–8.13
Tulathromycin						1.8	3.6	36.4	49.1	9.1	0	0				8	16	0–8.13
Tylosin tartrate					0	0	0	0	1.8	3.6	12.7	81.8				32	>32	0–8.13
Tildipirosin						96.4	3.6	0	0	0						1	1	0–8.13

^a^ The dilution ranges for the percentage of the isolates tested are those contained in the white area. Values above this range indicate MIC values higher than the highest concentration tested. Values corresponding to the lowest concentration tested indicated MIC values lower or equal to the lowest concentration within the range. When available, susceptible and resistance breakpoints are indicated in vertical green and red lines, respectively. Cut-off values were used according to CLSI document VET08. ^b^ Confidence interval based on % resistant.

**Table 4:** MIC distribution frequencies of *M. haemolytica* cattle isolates from Australia 2019.

	*Mannheimia haemolytica* (n = 33)																	
	MIC Distribution (µg/mL) ^a^															MIC_50_ (µg/mL)	MIC_90_ (µg/mL)	CI (95%) ^b^
Antibiotics	0.03	0.06	0.12	0.25	0.5	1	2	4	8	16	32	64	128	256	512			
Ampicillin				100	0	0	0	0	0	0						0.25	0.25	0–12.98
Ceftiofur				100	0	0	0	0	0							0.25	0.25	0–12.98
Clindamycin				0	0	0	0	6.1	63.6	30.3						8	16	0–12.98
Danofloxacin			100	0	0	0										0.12	0.12	0–12.98
Enrofloxacin			100	0	0	0	0									0.12	0.12	0–12.98
Florfenicol				0	97.0	3.0	0	0	0							0.5	0.5	0–12.98
Gamithromycin						90.9	6.1	0	0	3.0						1	2	0.2–17.5
Gentamicin						0	97.0	3.0	0	0						2	2	0–12.98
Neomycin								15.2	84.8	0	0					8	8	0–12.98
Tetracycline					93.9	6.1	0	0	0							0.5	0.5	0–12.98
Penicillin			75.8	24.2	0	0	0	0	0							0.12	0.25	0–12.98
Sulphadimethoxine														87.9	2.1	256	512	0–12.98
Spectinomycin									0	54.5	45.5	0				16	32	0–12.98
Tiamulin					0	0	0	0	12.1	60.6	27.3					16	32	0–12.98
Tilmicosin							6.1	27.3	60.6	3.0	3.0					4	8	0.2–17.5
Trimethoprim/sulfamethoxazole							100									2	2	0–12.98
Tulathromycin						0	0	0	97.0	0	0	0	3.0			8	8	0.2–17.5
Tylosin tartrate					0	0	0	0	0	0	15.2	84.8				32	≥64	0–12.98
Tildipirosin						84.8	12.1	3.0	0	0						1	1	0–12.98

^a^ The dilution ranges tested for the percentage of the isolates are those contained in the white area. Values above this range indicate MIC values higher than the highest concentration tested. Values corresponding to the lowest concentration tested indicated MIC values lower or equal to the lowest concentration within the range. When available, susceptible and resistance breakpoints are indicated in vertical green and red lines, respectively. Cut-off values were used according to CLSI document VET08.^b^ Confidence interval based on % resistant.

**Table 5:** MIC distribution frequencies of P. multocida cattle isolates from Australia 2014–2018.

	*Pasteurella multocida* (n = 75)																	
	MIC Distribution (µg/mL) ^a^															MIC_50_ (µg/mL)	MIC_90_ (µg/mL)	CI (95%) ^b^
Antibiotics	0.03	0.06	0.12	0.25	0.5	1	2	4	8	16	32	64	128	256	512			
Ampicillin				96.0	0	1.3	0	0	0	1.3	1.3					0.25	1	1.0–12.0
Ceftiofur				100	0	0	0	0	0							0.25	0.25	0–6.07
Clindamycin				0	0	0	2.7	2.6	16.0	34.7	44.0					16	>16	0–6.07
Danofloxacin			98.7	1.3	0	0										0.12	0.12	0–6.07
Enrofloxacin			98.7	1.3	0	0	0									0.12	0.12	0–6.07
Florfenicol				62.7	37.3	0	0	0	0							0.25	0.5	0–6.07
Gamithromycin						93.3	1.3	0	4.0	2.7						1	8	0.5–10.2
Gentamicin						22.7	53.3	22.7	0	1.3						2	4	0–6.07
Neomycin								54.7	33.3	8.0	0	4.0				8	16	0–6.07
Tetracycline					88.0	4.0	0	0	8.0							0.5	8	3.3–17.2
Penicillin			93.3	2.7	0	0	1.3	0	0	2.7						0.12	2	1.0–12.0
Sulphadimethoxine														100		256	256	0–6.07
Spectinomycin									10.7	52.0	37.3	0				16	32	0–6.07
Tiamulin					2.6	0	2.7	1.3	14.7	58.7	20.0					16	32	0–6.07
Tilmicosin							0	69.3	28.0	0	0	2.7				4	8	0.5–10.2
Trimethoprim/sulfamethoxazole							100									2	2	0–6.07
Tulathromycin						56.0	30.7	4.0	6.6	0	0	0	2.7			1	8	0.5–10.2
Tylosin tartrate					0	1.3	0	2.7	16.0	45.3	30.7	4.0				16	32	0–6.07
Tildipirosin						89.3	2.7	0	5.3	2.7						1	8	0–6.07

^a^ The dilution ranges tested are those contained in the white area. Values above this range indicate MIC values higher than the highest concentration tested. Values corresponding to the lowest concentration tested indicated MIC values lower or equal to the lowest concentration within the range. When available, susceptible and resistance breakpoints are indicated in vertical green and red lines, respectively. Cut-off values were used according to CLSI document VET08. ^b^ Confidence interval based on % resistant.

**Table 6:** MIC distribution frequencies of P. multocida cattle isolates from Australia 2019.

	*Pasteurella multocida* (n = 65)																	
	MIC Distribution (µg/mL) ^a^															MIC_50_ (µg/mL)	MIC_90_ (µg/mL)	CI (95%) ^b^
Antibiotics	0.03	0.06	0.12	0.25	0.5	1	2	4	8	16	32	64	128	256	512			
Ampicillin				95.4	0	0	0	0	1.5	3.1						0.25	0.5	1.2–13.8
Ceftiofur				100	0	0	0	0	0							0.25	0.25	0–6.95
Clindamycin				0	0	1.5	0	3.1	7.7	49.2	38.5					16	32	0–6.95
Danofloxacin			100	0	0	0										0.12	0.12	0–6.95
Enrofloxacin			100	0	0	0	0									0.12	0.12	0–6.95
Florfenicol				83.1	16.9	0	0	0	0							0.25	0.5	0–6.95
Gamithromycin						78.5	0	0	0	21.5	0					1	16	12.7–33.8
Gentamicin						16.9	69.2	12.3	1.5	0						2	4	0–6.95
Neomycin								67.7	13.8	18.5	0					4	8	0–6.95
Tetracycline					75.4	4.6	0	1.5	15.4	3.1						0.5	8	10.3–30.4
Penicillin			92.3	3.1	0	0	0	0	0	4.6						0.12	0.25	1.2–13.8
Sulphadimethoxine														100		256	256	0–6.95
Spectinomycin									10.8	64.6	24.6	0				16	32	0–6.95
Tiamulin					1.5	0	1.5	3.1	18.5	60.0	15.4					16	32	0–6.95
Tilmicosin							27.7	36.9	9.2	1.5	24.6					8	32	15.1–37.1
Trimethoprim/sulfamethoxazole							100									2	2	0–6.95
Tulathromycin						0	0	0	76.9	1.5	0	21.5				8	64	12.7–33.8
Tylosin tartrate					0	1.5	0	3.1	9.2	64.6	21.5					16	32	0–6.95
Tildipirosin						72.3	7.7	9.2	6.2	4.6	0					1	2	0–6.95

^a^ The dilution ranges tested for the percentage of the isolates are those contained in the white area. Values above this range indicate MIC values higher than the highest concentration tested. Values corresponding to the lowest concentration tested indicated MIC values lower or equal to the lowest concentration within the range. When available, susceptible and resistance breakpoints are indicated in vertical green and red lines, respectively. Cut-off values were used according to CLSI document VET08. ^b^ Confidence interval based on % resistant.

**Table 7:** Distribution of *Pasteurella multocida* isolates by year and resistance profile.

Year	Total Isolates	S	Tet-R	Mac-R	Tet-Mac-R	Pen-Amp-R	Amp-Pen-Tet-R	Amp-Pen-Tet-Mac-R
2014–2015	12	12	0	0	0	0	0	0
2016–2017	40	35	3	1 *	0	0	1	0
2018	23	19	1	1	0	1	1	0
2019	65	43	4	10	5	0	1	2 **
Total	140	109	8	12	5	1	3	2

S: susceptible, Tet-R: resistant to tetracyclines, Mac-R: resistant to the macrolides tilmicosin, tulathromycin, and gamithromycin, Pen-R: resistant to β-lactams, * This isolate was only resistant to tilmicosin and tulathromycin. ** These two MDR isolates were resistant to tilmicosin but remained susceptible to gamithromycin and tulathromycin.

**Table 8:** Resistance profile, RAPD pattern and presence of antimicrobial resistance genes among isolates of *Pasteurella multocida (P. m)* (*n* = 28) and *Mannheimia haemolytica (M. h)* (*n* = 1) +, present, −, absent.

CLN	ST	Year	RP	RAPD P	Antimicrobial Resistance Genes								
					*aph* *A1*	*bla*_ROB-1_	*msr*(E)	*mph*(E)	*strA*	*str* *B*	*sul* *2*	*tet*(Y)	*Tet*(H)-*tet*(R)
*P. m* 17BRD-035	QLD	2017	Amp, Pen and Tet	Cluster V	**−**	**+**	**−**	**−**	**−**	**−**	**−**	**−**	**+**
*P. m* 17BRD-041	QLD	2017	Tet	Cluster V	**+**	**−**	**−**	**−**	**+**	**+**	**+**	**+**	**−**
*P. m* 17BRD-042	QLD	2017	Tet	Cluster V	**+**	**−**	**−**	**−**	**+**	**+**	**+**	**+**	**−**
*P. m* 17BRD-038	VIC	2016	Tet	Cluster V	**+**	**−**	**−**	**−**	**+**	**+**	**+**	**+**	**−**
*P. m* 17BRD-039	QLD	2017	Tilm and Tul	Cluster V	**−**	**−**	**+**	**+**	**−**	**−**	**−**	**−**	**−**
*P.m* 18BRD-047	NSW	2018	Tet	Cluster V	**−**	**−**	**−**	**−**	**−**	**−**	**−**	**−**	**+**
*P. m* 18BRD-001	QLD	2018	Amp, Pen and Tet	Cluster V	**−**	**+**	**−**	**−**	**−**	**−**	**−**	**−**	**+**
*P. m* 18BRD-005	QLD	2018	Amp and Pen	Cluster V	**−**	**+**	**−**	**−**	**+**	**−**	**+**	**−**	**−**
*P. m* 18BRD-025	SA	2018	Til and Tul	Cluster V	**−**	**−**	**+**	**+**	**−**	**−**	**−**	**−**	**−**
*P. m* 19BRD-010	NSW	2019	Tet	Cluster VI	**−**	**−**	**−**	**−**	**−**	**−**	**−**	**−**	**+**
*P. m* 19BRD-011	NSW	2019	Gam, Til, Tul and Tet	Cluster VI	**−**	**−**	**+**	**+**	**−**	**−**	**−**	**−**	**+**
*P. m* 19BRD-014	NSW	2019	Tet	Cluster VI	**−**	**−**	**−**	**−**	**−**	**−**	**−**	**−**	**+**
*P. m*19BRD-016	SA	2019	Gam, Til and Tul	Cluster VI	**−**	**−**	**+**	**+**	**−**	**−**	**−**	**−**	**−**
*P. m* 19BRD-017	SA	2019	Gam, Til and Tul	Cluster VI	**−**	**−**	**+**	**+**	**−**	**−**	**−**	**−**	**−**
*P. m* 19BRD-020	SA	2019	Gam, Tiland Tul	Cluster VI	**−**	**−**	**+**	**+**	**−**	**−**	**−**	**−**	**−**
*P. m* 19BRD-032	QLD	2019	Amp, Pen, Tet and Til	Cluster VI	**−**	**+**	**−**	**−**	**−**	**−**	**−**	**−**	**+**
*P. m* 19BRD-039	QLD	2019	Gam, Til and Tul	Cluster VI	**−**	**−**	**+**	**+**	**−**	**−**	**−**	**−**	**−**
*P. m* 19BRD-042	QLD	2019	Amp, Pen and Tet	Cluster VII	**−**	**+**	**−**	**−**	**−**	**−**	**−**	**−**	**+**
*P. m* 19BRD-057	QLD	2019	Amp, Pen, Tet and Til	Cluster VIII	**−**	**+**	**−**	**−**	**−**	**−**	**−**	**−**	**+**
*P.m* 19BRD-085	NSW	2019	Gam, Til and Tul	Cluster IX	**−**	**−**	**+**	**+**	**−**	**−**	**−**	**−**	**−**
*P.m* 19BRD-094	NSW	2019	Gam, Til and Tul	Cluster IX	**−**	**−**	**+**	**+**	**−**	**−**	**−**	**−**	**−**
*P.m* 19BRD-098	NSW	2019	Gam, Til and Tul	Cluster IX	**−**	**−**	**+**	**+**	**−**	**−**	**−**	**−**	**−**
*P.m* 19BRD-100	NSW	2019	Gam, Til and Tul	Cluster VIII	**−**	**−**	**+**	**+**	**−**	**−**	**−**	**−**	**−**
*P.m* 19BRD-104	NSW	2019	Tet	Cluster VIII	**−**	**−**	**−**	**−**	**−**	**−**	**−**	**−**	**+**
*P.m* 19BRD-106	NSW	2019	Gam, Til, Tul and Tet	Cluster VIII	**−**	**−**	**+**	**+**	**−**	**−**		**−**	**+**
*P.m* 19BRD-110	NSW	2019	Gam, Til, Tul and Tet	Cluster VIII	**−**	**−**	**−**	**−**	**−**	**−**	**−**	**−**	**+**
*P.m* 19BRD-111	NSW	2019	Tet	Cluster VIII	**−**	**−**	**−**	**−**	**−**	**−**	**−**	**−**	**+**
*P. m* 19BRD-112	NSW	2019	Gam, Til, Tul and Tet	Cluster VIII	**−**	**−**	**+**	**+**	**−**	**−**	**−**	**−**	**+**
*P. m* 19BRD-141	NSW	2019	Tet	Cluster IV	**−**	**−**	**−**	**−**	**−**	**−**	**−**	**−**	**+**
*P. m* 19BRD-146	NSW	2019	Gam, Til Tul and Tet	Cluster IV	**−**	**−**	**−**	**−**	**−**	**−**	**−**	**−**	**+**
*M. h* 19BRD-084	NSW	2019	Gam, Til and Tul	Cluster III	**−**	**−**	**+**	**+**	**−**	**−**	**−**	**−**	**−**

CLN: clinical strains, RP: resistance profile, ST: State, RAPD P: RAPD Pattern, CLN: clinical strains, Amp: ampicillin; Gam: gamithromycin; Pen: penicillin; Tet: tetracycline, Til: tilmicosin; Tul: tulathromycin.

## Data Availability

The data presented in this study are available on request from the corresponding author. The data are not publicly available due to confidentiality issues.

## Supplementary Materials

The following are available online at https://www.mdpi.com/2076-2607/9/6/1322/s1, Figure S1, Random amplified polymorphic profiles and antimicrobial susceptibility profiles of 88 *M. haemolytica* strains. Figure S2, Random amplified polymorphic profiles and antimicrobial susceptibility profiles of 140 *P. multocida* strains.
