# Correction: Alhamami et al. First Emergence of Resistance to Macrolides and Tetracycline Identified in *Mannheimia haemolytica* and *Pasteurella multocida* Isolates from Beef Feedlots in Australia. *Microorganisms* 2021, *9*, 1322

**DOI:** 10.3390/microorganisms10040825

**Published:** 2022-04-15

**Authors:** Tamara Alhamami, Piklu Roy Chowdhury, Nancy Gomes, Mandi Carr, Tania Veltman, Manouchehr Khazandi, Joanne Mollinger, Ania T. Deutscher, Conny Turni, Layla Mahdi, Henrietta Venter, Sam Abraham, Steven P. Djordjevic, Darren J. Trott

**Affiliations:** 1 Australian Centre for Antimicrobial Resistance Ecology, School of Animal and Veterinary Sciences, University of Adelaide, Roseworthy Campus, Roseworthy, SA 5371, Australia; tamara.alhamami@adelaide.edu.au (T.A.); nancy.gomes@student.adelaide.edu.au (N.G.); tania.veltman@adelaide.edu.au (T.V.); khazandi@yahoo.com (M.K.); 2 The ithree Institute, University of Technology Sydney, City Campus, Ultimo, NSW 2007, Australia; piklu.bhattacharya@uts.edu.au (P.R.C.); steven.djordjevic@uts.edu.au (S.P.D.); 3 Department of Animal Health and Production, School of Animal and Veterinary Sciences, University of Adelaide, Roseworthy Campus, Roseworthy, SA 5371, Australia; mandi.carr@adelaide.edu.au; 4 Biosecurity Sciences Laboratory, Department of Agriculture and Fisheries, Coopers Plains, QLD 4108, Australia; joanne.mollinger@daf.qld.gov.au; 5 NSW Department of Primary Industries, Elizabeth Macarthur Agricultural Institute, Menangle, NSW 2568, Australia; ania.deutscher@dpi.nsw.gov.au; 6 Centre for Animal Science, Queensland Alliance for Agriculture and Food Innovation, The University of Queensland, St. Lucia, QLD 4072, Australia; c.turni1@uq.edu.au; 7 Clinical & Health Sciences, University of South Australia, Adelaide, SA 5000, Australia; layla.mahdi@unisa.edu.au (L.M.); rietie.venter@unisa.edu.au (H.V.); 8 Antimicrobial Resistance and Infectious Disease Laboratory, Murdoch University, Murdoch, WA 6150, Australia; s.abraham@murdoch.edu.au

The authors wish to make the following corrections to this paper [1]:

There are metadata errors in Supplementary Figure S2 and Table 8, which state that isolate 17BRD-035 was isolated from a feedlot in NSW when in fact it came from Queensland. Figure S2 in Supplementary Materials was changed accordingly and was included with a separate document: https://www.mdpi.com/article/10.3390/microorganisms9061322/s1

In Table 8, the ST column for strain *P. m* 17BRD-035 was changed from NSW to QLD, the correct Table 8 is as follows:

**Table 8:** Resistance profile, RAPD pattern and presence of antimicrobial resistance genes among isolates of *Pasteurella multocida (P. m)* (*n* = 28) and *Mannheimia haemolytica (M. h)* (*n* = 1) +, present, −, absent.

CLN	ST	Year	RP	RAPD P	Antimicrobial Resistance Genes								
					*aph* *A1*	*bla*_ROB-1_	*msr*(E)	*mph*(E)	*strA*	*str* *B*	*sul* *2*	*tet*(Y)	*Tet*(H)-*tet*(R)
*P. m* 17BRD-035	QLD	2017	Amp, Pen and Tet	Cluster V	**−**	**+**	**−**	**−**	**−**	**−**	**−**	**−**	**+**
*P. m* 17BRD-041	QLD	2017	Tet	Cluster V	**+**	**−**	**−**	**−**	**+**	**+**	**+**	**+**	**−**
*P. m* 17BRD-042	QLD	2017	Tet	Cluster V	**+**	**−**	**−**	**−**	**+**	**+**	**+**	**+**	**−**
*P. m* 17BRD-038	VIC	2016	Tet	Cluster V	**+**	**−**	**−**	**−**	**+**	**+**	**+**	**+**	**−**
*P. m* 17BRD-039	QLD	2017	Tilm and Tul	Cluster V	**−**	**−**	**+**	**+**	**−**	**−**	**−**	**−**	**−**
*P.m* 18BRD-047	NSW	2018	Tet	Cluster V	**−**	**−**	**−**	**−**	**−**	**−**	**−**	**−**	**+**
*P. m* 18BRD-001	QLD	2018	Amp, Pen and Tet	Cluster V	**−**	**+**	**−**	**−**	**−**	**−**	**−**	**−**	**+**
*P. m* 18BRD-005	QLD	2018	Amp and Pen	Cluster V	**−**	**+**	**−**	**−**	**+**	**−**	**+**	**−**	**−**
*P. m* 18BRD-025	SA	2018	Til and Tul	Cluster V	**−**	**−**	**+**	**+**	**−**	**−**	**−**	**−**	**−**
*P. m* 19BRD-010	NSW	2019	Tet	Cluster VI	**−**	**−**	**−**	**−**	**−**	**−**	**−**	**−**	**+**
*P. m* 19BRD-011	NSW	2019	Gam, Til, Tul and Tet	Cluster VI	**−**	**−**	**+**	**+**	**−**	**−**	**−**	**−**	**+**
*P. m* 19BRD-014	NSW	2019	Tet	Cluster VI	**−**	**−**	**−**	**−**	**−**	**−**	**−**	**−**	**+**
*P. m*19BRD-016	SA	2019	Gam, Til and Tul	Cluster VI	**−**	**−**	**+**	**+**	**−**	**−**	**−**	**−**	**−**
*P. m* 19BRD-017	SA	2019	Gam, Til and Tul	Cluster VI	**−**	**−**	**+**	**+**	**−**	**−**	**−**	**−**	**−**
*P. m* 19BRD-020	SA	2019	Gam, Tiland Tul	Cluster VI	**−**	**−**	**+**	**+**	**−**	**−**	**−**	**−**	**−**
*P. m* 19BRD-032	QLD	2019	Amp, Pen, Tet and Til	Cluster VI	**−**	**+**	**−**	**−**	**−**	**−**	**−**	**−**	**+**
*P. m* 19BRD-039	QLD	2019	Gam, Til and Tul	Cluster VI	**−**	**−**	**+**	**+**	**−**	**−**	**−**	**−**	**−**
*P. m* 19BRD-042	QLD	2019	Amp, Pen and Tet	Cluster VII	**−**	**+**	**−**	**−**	**−**	**−**	**−**	**−**	**+**
*P. m* 19BRD-057	QLD	2019	Amp, Pen, Tet and Til	Cluster VIII	**−**	**+**	**−**	**−**	**−**	**−**	**−**	**−**	**+**
*P.m* 19BRD-085	NSW	2019	Gam, Til and Tul	Cluster IX	**−**	**−**	**+**	**+**	**−**	**−**	**−**	**−**	**−**
*P.m* 19BRD-094	NSW	2019	Gam, Til and Tul	Cluster IX	**−**	**−**	**+**	**+**	**−**	**−**	**−**	**−**	**−**
*P.m* 19BRD-098	NSW	2019	Gam, Til and Tul	Cluster IX	**−**	**−**	**+**	**+**	**−**	**−**	**−**	**−**	**−**
*P.m* 19BRD-100	NSW	2019	Gam, Til and Tul	Cluster VIII	**−**	**−**	**+**	**+**	**−**	**−**	**−**	**−**	**−**
*P.m* 19BRD-104	NSW	2019	Tet	Cluster VIII	**−**	**−**	**−**	**−**	**−**	**−**	**−**	**−**	**+**
*P.m* 19BRD-106	NSW	2019	Gam, Til, Tul and Tet	Cluster VIII	**−**	**−**	**+**	**+**	**−**	**−**		**−**	**+**
*P.m* 19BRD-110	NSW	2019	Gam, Til, Tul and Tet	Cluster VIII	**−**	**−**	**−**	**−**	**−**	**−**	**−**	**−**	**+**
*P.m* 19BRD-111	NSW	2019	Tet	Cluster VIII	**−**	**−**	**−**	**−**	**−**	**−**	**−**	**−**	**+**
*P. m* 19BRD-112	NSW	2019	Gam, Til, Tul and Tet	Cluster VIII	**−**	**−**	**+**	**+**	**−**	**−**	**−**	**−**	**+**
*P. m* 19BRD-141	NSW	2019	Tet	Cluster IV	**−**	**−**	**−**	**−**	**−**	**−**	**−**	**−**	**+**
*P. m* 19BRD-146	NSW	2019	Gam, Til Tul and Tet	Cluster IV	**−**	**−**	**−**	**−**	**−**	**−**	**−**	**−**	**+**
*M. h* 19BRD-084	NSW	2019	Gam, Til and Tul	Cluster III	**−**	**−**	**+**	**+**	**−**	**−**	**−**	**−**	**−**

CLN: clinical strains, RP: resistance profile, ST: State, RAPD P: RAPD Pattern, CLN: clinical strains, Amp: ampicillin; Gam: gamithromycin; Pen: penicillin; Tet: tetracycline, Til: tilmicosin; Tul: tulathromycin.

The authors apologize for any inconvenience caused and state that the scientific conclusions are unaffected. The original article has been updated.
