# Supplementary material for: First Emergence of Resistance to Macrolides and Tetracycline Identified in Mannheimia haemolytica and Pasteurella multocida Isolates from Beef Feedlots in Australia
Source: Microorganisms. 2021 Jun 17;9(6):1322. doi: 10.3390/microorganisms9061322 (PMC8233904; doi:10.3390/microorganisms9061322)
Supplement: Supplementary file 1 [file microorganisms-09-01322-s001.zip › microorganisms-1240085-updated supplementary.pdf]

Supplementary Material:

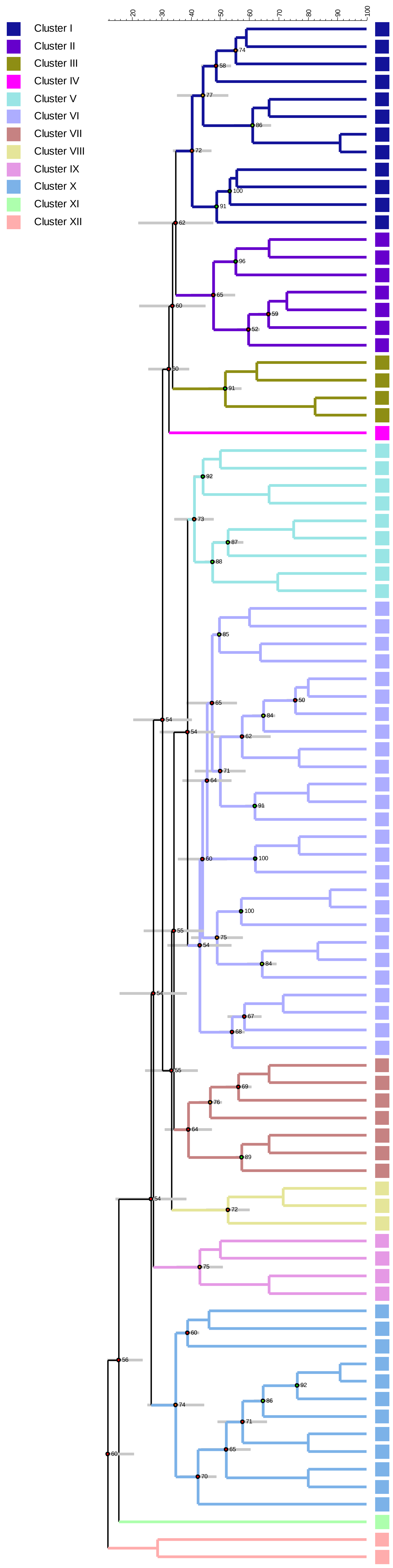

Cluster	State	MDR	Isolate ID
Cluster I	QLD		17BRD-013
Cluster I	NSW		17BRD-016
Cluster I	QLD		18BRD-003
Cluster I	QLD		17BRD-069
Cluster I	QLD		17BRD-074
Cluster I	QLD		17BRD-078
Cluster I	QLD		17BRD-075
Cluster I	QLD		17BRD-080
Cluster I	QLD		17BRD-011
Cluster I	NSW		18BRD-038
Cluster I	QLD		17BRD-012
Cluster I	QLD		17BRD-079
Cluster II	QLD		17BRD-073
Cluster II	QLD		17BRD-077
Cluster II	QLD		17BRD-072
Cluster II	QLD		17BRD-015
Cluster II	QLD		19BRD-027
Cluster II	QLD		17BRD-068
Cluster II	QLD		17BRD-070
Cluster III	QLD		19BRD-044
Cluster III	NSW	Tulth,TILMIC and GAM	19BRD-084
Cluster III	NSW		18BRD-040
Cluster III	NSW		18BRD-041
Cluster IV	QLD		19BRD-051
Cluster V	QLD		17BRD-007
Cluster V	QLD		17BRD-009
Cluster V	QLD		17BRD-003
Cluster V	QLD		17BRD-004
Cluster V	QLD		19BRD-076
Cluster V	QLD		19BRD-078
Cluster V	QLD		17BRD-001
Cluster V	QLD		18BRD-016
Cluster V	QLD		18BRD-017
Cluster VI	QLD		18BRD-011
Cluster VI	NSW		19BRD-160
Cluster VI	NSW		17BRD-071
Cluster VI	QLD		19BRD-161
Cluster VI	QLD		19BRD-170
Cluster VI	QLD		19BRD-181
Cluster VI	QLD		19BRD-189
Cluster VI	QLD		19BRD-172
Cluster VI	QLD		19BRD-163
Cluster VI	QLD		19BRD-186
Cluster VI	QLD		18BRD-004
Cluster VI	QLD		19BRD-185
Cluster VI	SA		18BRD-024
Cluster VI	QLD		18BRD-013
Cluster VI	QLD		18BRD-015
Cluster VI	NSW		18BRD-034
Cluster VI	SA		19BRD-002
Cluster VI	SA		19BRD-006
Cluster VI	NSW		18BRD-035
Cluster VI	SA		18BRD-023
Cluster VI	NSW		18BRD-032
Cluster VI	QLD		17BRD-006
Cluster VI	NSW		18BRD-033
Cluster VI	NSW		18BRD-036
Cluster VI	NSW		18BRD-037
Cluster VI	SA		19BRD-019
Cluster VII	QLD		19BRD-187
Cluster VII	QLD		19BRD-188
Cluster VII	NSW		17BRD-017
Cluster VII	NSW		17BRD-014
Cluster VII	QLD		17BRD-008
Cluster VII	QLD		17BRD-019
Cluster VII	QLD		17BRD-076
Cluster VIII	QLD		17BRD-018
Cluster VIII	QLD		17BRD-020
Cluster VIII	VIC		17BRD-065
Cluster IX	QLD		17BRD-002
Cluster IX	QLD		19BRD-045
Cluster IX	QLD		18BRD-002
Cluster IX	SA		19BRD-114
Cluster X	QLD		19BRD-052
Cluster X	QLD		19BRD-071
Cluster X	QLD		18BRD-009
Cluster X	NSW		19BRD-117
Cluster X	NSW		19BRD-118
Cluster X	SA		19BRD-115
Cluster X	QLD		17BRD-010
Cluster X	NSW		19BRD-121
Cluster X	NSW		19BRD-122
Cluster X	NSW		19BRD-119
Cluster X	NSW		19BRD-120
Cluster X	NSW		19BRD-123
Cluster XI	VIC		17BRD-067
Cluster XII	NSW		18BRD-039
Cluster XII	NSW		19BRD-012

Figure S1. Random amplified polymorphic profiles and antimicrobial susceptibility profiles of 88 *M. haemolytica* strains. The dendrogram was generated with BioNumerics (Applied Maths) based on the Dice coefficient. The cluster number, Australian state of origin (NSW, New South Wales; QLD, Queensland; VIC, Victoria; SA, South Australia), multidrug-resistance (MDR) status (Tulth, tulthromycin, TLMIC, tilmicosin and GAM, gamithromycin resistant) and isolate identification number, are from right to left side, respectively.

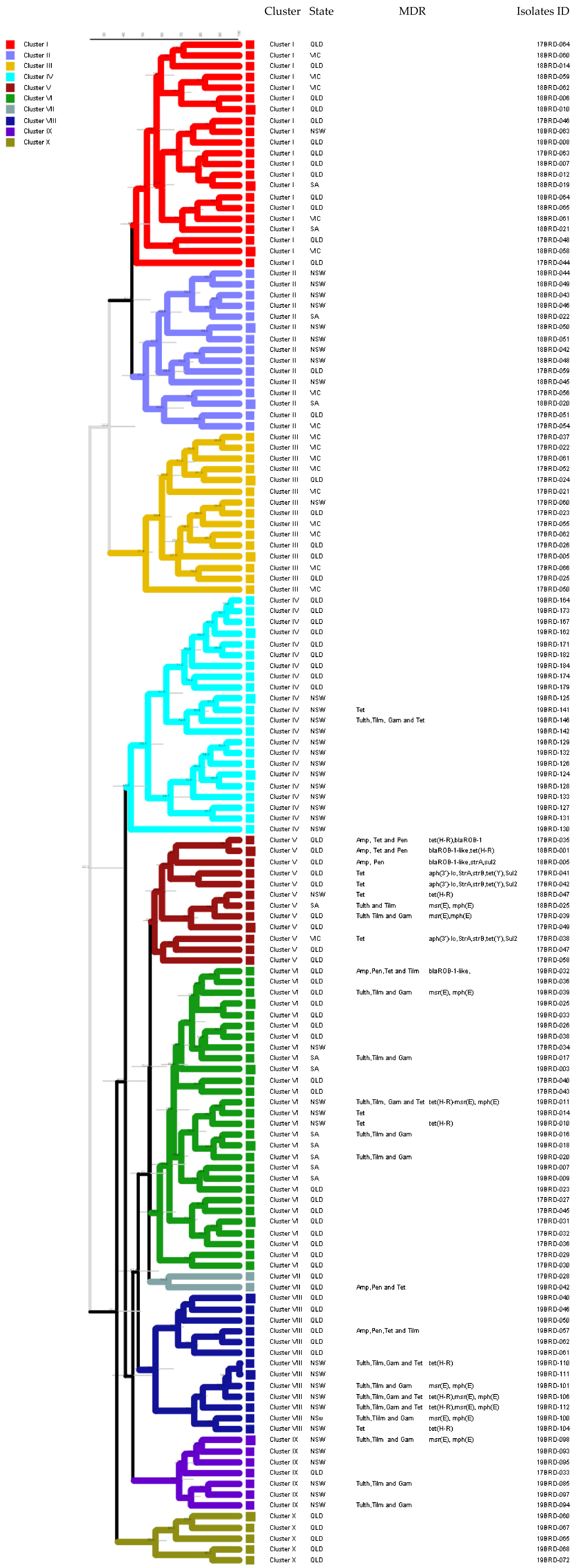

Figure S2. Random amplified polymorphic profiles and antimicrobial susceptibility profiles and antimicrobial susceptibility profiles of 140 *P. multocida* strains. The dendrogram was generated with BioNumerics program (Applied Maths) based on the Dice coefficient. The cluster number, Australian state of origin (NSW, New South Wales; QLD, Queensland; VIC, Victoria; SA, South Australia), multidrug-resistance (MDR) status (Tulth, tulthromycin, TLMIC, tilmicosin, GAM, gamithromycin, AMP, ampcilin and Pen, penicillin resistant) and isolate identification number, are from right to left side, respectively.
